# Time to lithium: A case register study of lithium initiation in bipolar disorder

**DOI:** 10.1177/02698811251408749

**Published:** 2026-02-13

**Authors:** Violeta Perez-Rodriguez, Timothy Nguyen, Katherine Beck, Emma Butler, Rashmi Patel, Rebecca Strawbridge, Oliver Howes, Allan H. Young, Sameer Jauhar

**Affiliations:** 1 Department of Psychosis Studies, Institute of Psychiatry Psychology & Neuroscience, King’s College London, UK; 2 TREAT Service, South London and Maudsley NHS Foundation Trust, UK; 3 Schmack Lab, The Francis Crick Institute, London, UK; 4 Department of Psychiatry, University of Cambridge, UK; 5 Centre for Affective Disorders, Institute of Psychiatry Psychology & Neuroscience, King’s College London, UK; 6 Department of Brain Sciences, Imperial College London, UK

**Keywords:** bipolar disorder, lithium, treatment, early intervention, electronic health records

## Abstract

**Background:**

Lithium monotherapy has been recommended as first-line maintenance or long-term pharmacological therapy for bipolar disorder (BD). Previous research has linked early lithium use with better outcomes for people with BD. Despite extensive evidence as an effective treatment for BD, lithium prescribing continues to decline.

**Aims:**

Our primary aim was to determine the time between initial assessment by mental health services and lithium initiation in people with BD who were prescribed and concordant with lithium. Secondary objectives included determining the time between the first assessment and recorded BD diagnosis, number of prior mood episodes, polarity when lithium was prescribed and number of antipsychotics before lithium initiation.

**Methods:**

Free-text clinical notes were extracted from a de-identified electronic health record database. Eligible records comprised adults with a BD diagnosis who were concordant with lithium treatment.

**Results:**

Eighty-eight people were identified, based on inclusion and exclusion criteria. Median time between first assessment and lithium initiation was 659 days. Median time between first assessment and BD diagnosis was 220 days, with a median of 2.5 mood episodes and 2 antipsychotics prescribed prior to lithium. Around 30% of people presented with manic symptoms at the time of lithium prescription. There is a significant delay between first contact with services and initiation of lithium in people with BD.

**Conclusions:**

This highlights the potential for earlier intervention with lithium, which could improve outcomes for people with BD.

## Background

Lithium monotherapy is recommended as first-line maintenance treatment for bipolar disorder (BD) by all major international guideline groups, including the British Association for Psychopharmacology (Goodwin et al., 2016), UK National Institute for Health and Care Excellence guidelines (NICE, 2023), the Canadian Network for Mood and Anxiety Treatments (Keramatian et al., 2023), The International College of Neuro-Psychopharmacology (Fountoulakis et al., 2016), the Royal Australian and New Zealand College of Psychiatrists (Malhi et al., 2021), the American Psychiatric Association (APA, 2002) and the World Federation of Societies of Biological Psychiatry (Grunze et al., 2002). This is unsurprising, given existing evidence for its efficacy in prophylaxis of mood episodes of both polarities, similarly to other compounds such as quetiapine (Nestsiarovich et al., 2022). Furthermore, lithium has also been linked to the prevention of completed suicide in mood disorders (Del Matto et al., 2020).

Use of lithium in early BD has been examined in a randomised controlled trial in first episode mania, comparing lithium to quetiapine monotherapy and finding improved symptom scores and functioning in the lithium group (Berk et al., 2017). A Danish case register study suggested early lithium use, defined as the start of lithium after the first contact or a single mood episode, is related to better outcomes than later use (Kessing et al., 2014). A recent systematic review by the International Society for Bipolar Disorders (ISBD) Taskforce on early intervention found that treatment with mood stabilisers was linked to better global functioning compared to antipsychotics, and lithium led to lower recurrence risk compared to other mood stabilisers (Ratheesh et al., 2023), although the existing evidence base is limited. These lines of evidence suggest early use of lithium is effective and guidelines reflect this (Chia et al., 2019).

Despite this, lithium use has declined in clinical practice (Kessing, 2024; Lyall et al., 2019; Rhee et al., 2020). Potential reasons include concerns about side effect profile and tolerability (Hidalgo-Mazzei et al., 2023), diminished confidence amongst clinicians (Malhi et al., 2023), widespread use of second-generation antipsychotics which are often used instead of lithium for maintenance treatment (Jauhar and Young, 2019), and lack of commercial support due to not having a medication patent (Malhi et al., 2020). Interestingly, a recent survey carried out by the ISBD in collaboration with the International Group for the Study of Lithium-Treated Patients found contextual factors negatively impacted clinicians’ attitudes towards lithium, such as practicing in the private sector or in developing countries. They hypothesised that limited resources for monitoring, or costs of such tests when resources are available, could explain these results (Hidalgo-Mazzei et al., 2023).

There has therefore been renewed focus on earlier lithium initiation in people with BD (Malhi and Bauer, 2023; Malhi et al., 2023). Whilst a recent meta-analysis found significant delay between symptom onset and evidence-based interventions such as mood stabilisers (Scott et al., 2022), we are unaware of any studies examining time to initiation of lithium, diagnosis of BD and prior treatment, in cohorts of people with BD.

The primary aim of this study was to determine the time between BD diagnosis and initial lithium prescription. Secondary objectives included determining the following: (a) time between first assessment and recorded diagnosis of BD, (b) number of mood episodes experienced by those with BD before lithium was prescribed, (c) predominant mood polarity when lithium was prescribed, and (d) number of antipsychotics and mood stabilisers prescribed before lithium was initiated.

## Methods

### Ethical approval

The study was approved by the Clinical Records Interactive Search (CRIS) Oversight Committee in November 2020. CRIS has ethical approval from the South Central – Oxford C Research Ethics Committee (REC) as a database for secondary research. The REC reference is: 23/SC/0257.

### Participants

Free-text clinical notes were extracted from the South London and Maudsley NHS Foundation Trust (SLaM) Biomedical Research Centre CRIS, which comprises a de-identified electronic health record database established in 2008 of a public secondary mental health service covering a catchment population of approximately 1.3 million people in South London, UK (Perera et al., 2016). Inclusion criteria were as follows: (a) having an ICD-10 diagnosis of F30 (Manic episode) or F31 (Bipolar affective disorder) recorded between 1 January 2015 and 1 January 2020, (b) age 18 years on date of the first recorded F30 or F31 diagnosis, (c) having had at least one face-to-face assessment with a medically qualified practitioner, (d) receiving treatment with lithium at therapeutic dose, ascertained by a laboratory recorded lithium serum level above 0.0 mmol/L, (e) having a continuous first episode of care within SLaM for at least 6 months during which the F30 or F31 diagnosis was recorded on fixed fields. The arbitrary inclusion criterion that the patient had received care for at least 6 months was used to ensure services had sufficient time to establish a diagnosis of BD and discuss treatment options, including lithium, with the patient.

Exclusion criteria included (a) any recorded treatment or episode of care outside of SLaM before lithium prescription, or (b) unclear or incomplete clinical documentation. Data were extracted on 16 February 2021, and free-text clinical notes were chronologically read line-by-line by a psychiatrist (author T.N.). Language processing or other data algorithms were not used.

### Measures

Assessments of age, gender, ethnicity, and length of illness were recorded from the date of data extraction. Physical health comorbidities (hypertension, diabetes, thyroid disorder, renal disorder or other), smoking status, alcohol dependence and illicit substance misuse were ascertained through manual review of clinical notes. Substance misuse was ascertained if clinical notes indicated a clinician-recorded history of misuse or if there was at least one documented positive urine drug screen during the episode of care.

Dates of first face-to-face assessment, when a diagnosis of BD was recorded, when lithium was considered or discussed with the patient, and when lithium was prescribed were manually reviewed and recorded from reading the free-text clinical notes. The number of mood episodes before lithium was prescribed was also recorded when a clinical impression of “depressed,” “mixed,” “hypomanic” or “manic” mood was recorded in the notes for more than a month. The rationale for choosing this timeframe was the temporal inaccuracy of clinical notes, where mention of symptoms within notes can continue beyond the conclusion of a clinical presentation, making the notes reflect this for longer than the actual symptoms. On the other hand, having a timeframe that is too long for symptoms to be considered as an “episode” might lead to our search missing some participants with shorter episodes. Similar approaches have been suggested in previous CRIS research to solve this problem (Kadra et al., 2015). A euthymic mood state lasting longer than 1 month was required to differentiate mood states from each other and was defined by a documented clinical impression of euthymia, normal mood, or the explicit end to the disordered mood state. A psychotic episode was determined by clinical notes indicating the patient had psychosis at any assessment. The number of inpatient admissions, antipsychotics, antidepressants and mood stabilisers other than lithium (lamotrigine, valproate, carbamazepine) were also recorded if these medications were prescribed at therapeutic doses, according to the British National Formulary (version BNF80; BMJ Group, 2020). The first ICD-10 diagnosis recorded by a clinician on the first face-to-face assessment, often documented as a provisional impression, was also recorded. Descriptive calculations were performed in Microsoft Excel (version 16 for Mac, developed by Microsoft Corporation).

## Results

A total of 213 people were identified through CRIS using the inclusion criteria, and 125 (58.6%) were excluded after manual review of clinical notes. Most exclusions were due to incomplete or unclear documentation of events not allowing to ascertain of when a diagnosis was first given or when lithium was initiated (41 people), a recorded episode of care with another mental health Trust (38 people) or receipt of care outside of the UK or from a private psychiatrist (13 patients). We also excluded those given a BD diagnosis before CRIS records began in 2008 (24 patients), and those who had an amended diagnosis other than BD when lithium was prescribed (5 patients) or opted out of CRIS (4 patients).

The included cohort (88 people; see Table 1) was primarily female (69.3%), had a mean age of 34.8 years (Standard deviation (SD) 12.0) and a mean length of follow-up of 5 years (SD 2.9). Many patients (40.9% of the total) had a recorded lifetime history of cigarette smoking, as well as a recorded history of illicit substance use. Full demographic characteristics of our sample are described in Table 1.

**Table 1.** Demographic details.

Characteristic frequency	
Age (years)	
Mean (SD)	34.8 (12)
Median (IQR)	31 (15)
Sex, *n* (%)	
Female	61 (69.3)
Male	27 (30.7)
Length of follow-up (years)	
Mean (SD)	5 (2.9)
Median (IQR)	5 (4)
Ethnicity, *n* (%)	
White	38 (43.2)
Black	25 (28.4)
Asian	9 (10.2)
Mixed	5 (5.7)
Other	7 (8)
Not recorded	4 (4.5)
History of substance use, *n* (%)	
Tobacco	36 (40.9)
Alcohol dependence	4 (4.5)
Cannabis	69 (78.4)
Cocaine	42 (47.7)
MDMA	15 (17.0)
Co-morbidity	
Hypertension	3 (3.4)
Diabetes	2 (2.3)
Thyroid disorder	6 (6.8)
Other	7 (8)

IQR: interquartile range; SD: standard deviation; MDMA: 3,4-Methylenedioxymethamphetamine.

### Time between assessment and lithium therapy

Time between assessment and key events, including lithium prescription, has been detailed in Table 2. Median time between first face-to-face assessment by a medically qualified practitioner within SLaM and recorded BD diagnosis was 220 days (Interquartile range (IQR) 1052). Median total time delay from the first assessment and prescribing of lithium was 659 days (IQR 1346).

**Table 2.** Time between assessment and key events.

Time (days) between	Mean (SD)	Median (IQR; Q1–Q3)
Assessment by a medic and diagnosis of BD	673 (891)	220 (1052; 35–1087)
Diagnosis of BD and consideration of lithium	214 (380)	25 (260; 0–260)
Consideration of lithium and prescription of lithium	157 (439)	3 (68; 0–68)
Assessment and prescription of lithium	1043 (1015.14)	659 (1346; 149–1496)

IQR: interquartile range; SD: standard deviation; BD: bipolar disorder.

Zero days was assumed in five cases where explicit consideration of lithium was not documented prior to lithium prescription. This time period (0 days) was also assumed in 30 cases where lithium was considered and prescribed to treat a mood state before a formal BD diagnosis was documented. This has led to a significant difference between the median and the mean, as noted above. A separate sensitivity analysis excluding patients for whom a time of 0 days between diagnosis and lithium was assumed was conducted, finding that the mean time between initial assessment and prescription of lithium was 1138 days (SD 1006) and the median time from assessment to lithium was 912 days (IQR 1355).

Given that a significant part of the cohort did not receive an initial diagnosis of BD, we conducted a sensitivity analysis including only those participants who received initial diagnoses of BD or mania. Mean time between initial assessment and prescription of lithium was 461 days (SD 552), and median time from assessment to lithium was 240 days (IQR 717). As such, we still found a significant delay in the prescription of lithium for those with an initial diagnosis of BD, although smaller than compared to the whole cohort.

### Events preceding lithium prescription

Initial diagnoses have been recorded in Table 3. The most common initial diagnosis given was mania (24 people, 27.3% of the total sample), followed by BD (18 people, 20.5%) and unipolar depressive disorders (9 people, 10.2%).

**Table 3.** Initial diagnoses.

Initial diagnosis	*n*	%
Mania	24	27.3
BD	18	20.4
Unipolar depressive disorders	9	10.2
Anxiety disorders (including OCD)	9	10.2
Substance-induced disorders	7	7.9
Schizophrenia spectrum disorders	11	12.5
Personality disorders	1	1.2
Other	9	10.2

BD: bipolar disorder; OCD: obsessive-compulsive disorder.

### Mood state and treatment setting at time of lithium prescription

Events preceding lithium prescription have been described in Table 4. When lithium was prescribed, 50% of the cohort was experiencing mania, and 40 patients (45.5%) were experiencing psychotic symptoms. Lithium was most commonly initiated in an inpatient setting (60 patients, 68.2% of cases).

**Table 4.** Events preceding lithium prescription.

Event preceding lithium prescription	Mean (SD)	Median (IQR)
Number of preceding mood episodes		
Any	2.6 (1.5)	2.5 (1)
Hypomanic/manic	1.3 (1.1)	1 (1)
Mixed	0.3 (0.6)	0 (0)
Depressive	1 (1.2)	1 (0)
Number of preceding psychotic episodes	0.9 (0.8)	1 (0)
Number of inpatient admissions	1.7 (1.4)	1 (1)
Number of antipsychotics prescribed	2.0 (2.1)	2 (2)

IQR: interquartile range; SD: standard deviation.

The median number of mood episodes before lithium prescription was 2.5 (IQR 3), and 40 people within the cohort (45.5%) had been prescribed at least 1 antidepressant prior to lithium. The median number of antipsychotics prescribed before lithium was two. Thirty individuals (34.1%) had been prescribed at least one other mood stabiliser prior to lithium.

## Discussion

To the best of our knowledge, this is the first study to examine, in a geographically defined cohort, the time between initial presentation to secondary services and prescription of lithium. We detailed the clinical characteristics of lithium prescription in a cohort of people diagnosed with BD in a large secondary mental health service in the UK. Most commonly (50% of cases), people were prescribed lithium for treatment of mania, and most people (68.2%) were prescribed lithium in an inpatient setting after a median prescription of two different antipsychotics.

There was a long delay between initial assessment with services and prescription of lithium (median 659 days). People presented with a median of 2.5 mood episodes prior to lithium being prescribed. Patients had received other medication prior to lithium, including a median of two antipsychotics and at least one other mood stabiliser in about 34% of the cases.

An important factor in delayed prescription of lithium was the delay in receiving a diagnosis of BD after initial assessment with a doctor, the median lapse being 220 days (IQR 1052; range 35–1087 days). This delay in receiving a diagnosis has previously been identified and associated with a delay in treatment for BD (Scott et al., 2022).

A relevant aspect potentially linked to the diagnostic delay could be the high prevalence of substance misuse, highlighted in previous studies (Messer et al., 2017). Although our numbers highlight lifetime use not necessarily occurring at the time of assessment, such comorbidity could contribute to diagnostic uncertainty.

Interestingly, our sample was comprised of a significant majority of female patients (69.3% vs. 30.7% of males). At the time of the review of clinical records, valproate prescription was significantly limited in women of childbearing age, given its known teratogenic effect (Valentino et al., 2024). Such restrictions have since been extended to males (MHRA, 2023), but this was not the case at the time of our clinical record review. Given that our sample is comprised of individuals who were prescribed and compliant with lithium, there could be a preference to prescribe other mood stabilisers such as valproate in male patients, leading to an under-representation of such population in our sample.

The main strength of our study is that data were obtained directly from clinical information recorded by staff working in our Trust. This makes the data more generalisable as an accurate representation of clinical practice within this setting (secondary services in the UK). In addition, the clinical records of all 88 patients have been hand-searched to obtain the information provided in the article, which is likely to maximise accuracy.

Some limitations should be acknowledged. Firstly, the sample is relatively small (88 individuals) and included participants from only one Mental Health Trust, and may therefore not be wholly representative of the general population of people with BD. Secondly, the cohort only included patients who were adherent to lithium treatment, making our results somewhat less extrapolable to a general clinical population. Thirdly, although people with previous treatment episodes outside of our Trust were excluded, some of these episodes may not have been recorded. In such cases, more pharmacological interventions could have been prescribed prior to treatment within our Mental Health Trust, and the delay in receiving a diagnosis of BD could be longer than our study suggests. This has been found in studies conducted using electronic mental health records (Patel et al., 2015) and should be noted when interpreting the results. Our search was also retrospective, and no events or outcomes after the prescription of lithium were collected.

One limitation of our inclusion criteria was that patients with shorter durations of care were excluded, which may have inflated estimates of the time until lithium was initiated, though the length of time in the general sample argues against this. This will be worth addressing in future prospective studies. Ideally, we would have included all those people for whom lithium was potentially indicated, though the methodology (mention of lithium in notes) precluded this. Furthermore, BD diagnoses were often recorded after lithium had already been prescribed. This likely reflects clinicians prioritising patient care over immediate documentation in the electronic health record. As a result, the interval between BD diagnosis and lithium prescription may appear artificially shortened.

Similarly, the timeframe chosen to define mood episodes (1 month) was selected based on our clinical experience in reviewing clinical records and the known temporal inaccuracy of clinical record notes. However, we are aware that any time limitation could artificially reduce or increase the number of mood episodes presented. There is no available consensus on how to manage this limitation or what time window to consider, and we based our decision on previous available literature suggesting a similar approach (Kadra et al., 2015).

Diagnosis was made through structured fields, which are more likely to be filled out in inpatient settings, given Key Performance Indicator requirements in such settings. This is a possible confounding factor, making individuals in our sample more likely to be prescribed lithium in an inpatient environment. This is balanced by the fact that mania, the cardinal feature of bipolar disorder, will be diagnosed in an inpatient setting, and this tends to provoke the prescribing of lithium. This decision was made to ensure that the final diagnosis, rather than a provisional or working diagnosis, was recorded.

Our findings underscore a critical gap between clinical guidelines and real-world prescribing: much like clozapine in treatment-resistant schizophrenia (Howes et al., 2012; Kane et al., 2019), lithium remains underutilised despite strong evidence and guideline support. Addressing this lag in implementation should be a priority for improving long-term outcomes in bipolar disorder.
